# Swab and Sputum SARS-CoV-2 RNA-Negative, CT-Positive, Symptomatic Contacts of COVID-19 Cases: A Hypothesis-Generating Prospective Population-Based Cohort Study of Eight Clusters

**DOI:** 10.3389/fmed.2021.685544

**Published:** 2021-08-17

**Authors:** Lei Huang, Xiuwen Zhang, Lingli Zhang, Jingjing Xu, Zhijian Wei, Yuanhong Xu, Chengyuan Zhang, Aman Xu

**Affiliations:** ^1^Department of Oncology, Ruijin Hospital, Shanghai Jiao Tong University School of Medicine, Shanghai, China; ^2^Department of General Surgery, The First Affiliated Hospital of Anhui Medical University, Hefei, China; ^3^Quarantine Ward for Respiratory Infectious Diseases, Feidong People's Hospital, East District of the First Affiliated Hospital of Anhui Medical University, Hefei, China; ^4^Graduate School, Soochow University, Suzhou, China; ^5^Department of Clinical Laboratory Medicine, The First Affiliated Hospital of Anhui Medical University, Hefei, China; ^6^Department of Respiratory and Critical Care Medicine, Feidong People's Hospital, East District of the First Affiliated Hospital of Anhui Medical University, Hefei, China

**Keywords:** COVID-19, SARS-CoV-2, RNA negative CT positive symptomatic contacts, confirmed COVID-19 cases, non-COVID-19 pneumonia cases, case quarantine and contact tracing, prospective population-based cohort study, multi-cluster study

## Abstract

**Background:** While some contacts of COVID-19 cases become symptomatic and radiographically abnormal, their SARS-CoV-2 RNA tests remain negative throughout the disease course. This prospective population-based cohort study aimed to explore their characteristics and significances.

**Methods:** From January 22, 2020, when the first COVID-19 case was identified in Hefei, China, until July 3, a total of 14,839 people in Feidong, Hefei, with a population of ~1,081,000 underwent SARS-CoV-2 RNA testing, where 36 cases (0.2%) with confirmed COVID-19 infection (Group 1) and 27 close contacts (0.2%) testing negative for SARS-CoV-2 RNA but having both positive COVID-19 exposure histories and CT findings (Group 2) from eight clusters were prospectively identified. Another 62 non-COVID-19 pneumonia cases without any exposure history (Group 3) were enrolled, and characteristics of the three groups were described and compared. We further described a cluster with an unusual transmission pattern.

**Results:** Fever was more common in Group 2 than Groups 1 and 3. Frequency of diarrhea in Group 1 was higher than in Groups 2 and 3. Median leucocyte, neutrophil, monocyte, and eosinophil counts were all lower in Groups 1 and 2 than in Group 3. Median D-dimer level was lower in Group 1 than in Groups 2 and 3. Total protein and albumin levels were higher in Groups 1 and 2 than in Group 3. C-reactive protein level was lower and erythrocyte sedimentation rate slower in Groups 1 and 2 than in Group 3. Combination antibacterial therapy and levofloxacin were more often used in Group 3 than in Groups 1 and 2. Lopinavir/ritonavir was more often administered in Groups 1 and 2 than in Group 3. Group 1 received more often corticosteroids than Groups 2 and 3. Group 2 received less often oxygen therapy than Groups 1 and 3. Median duration from illness onset to discharge was longer in Group 1 (27 d) than Groups 2 and 3 (both 17 d). Among contacts of a confirmed COVID-19 patient, only one had a positive virus RNA test but remained asymptomatic and had negative CT findings, and three had negative virus RNA tests but had symptoms and positive CT findings, one of whom transmitted COVID-19 to another asymptomatic laboratory-confirmed patient who had no other exposures.

**Conclusions:** Among close contacts of confirmed COVID-19 cases, some present with positive symptoms and CT findings but test negative for SARS-CoV-2 RNA using common respiratory (throat swab and sputum) specimens; they have features more similar to confirmed COVID-19 cases than non-COVID-19 pneumonia cases and might have transmitted SARS-CoV-2 to others. Such cases might add to the complexity and difficulty of COVID-19 control. Our hypothesis-generating study might suggest that SARS-CoV-2 RNA testing by rRT-PCR assays of common respiratory (throat swab and sputum) specimens alone, the widely accepted “golden standard” for diagnosing COVID-19, might be sometimes insufficient, and that further studies with some further procedures (e.g., testing via bronchoalveolar lavage or specific antibodies) would be warranted for Group 2-like patients, namely, the SARS-CoV-2 RNA-negative (tested using common respiratory specimens), radiographically positive, symptomatic contacts of COVID-19 cases, to further reveal their nature.

## Background

COVID-19 caused by SARS-CoV-2 has been quickly spreading around the world causing a pandemic (1). SARS-CoV-2 appears cunning and it seems challenging to wipe it out. Despite continued efforts of strict COVID-19 control measures including case quarantine and contact tracing, some places are experiencing second attack waves with increasing COVID-19 incidence again after preliminary control (1, 2).

SARS-CoV-2 RNA test is regarded the gold standard of diagnosing COVID-19. Notably, some close contacts of laboratory-confirmed COVID-19 cases become symptomatic and radiographically positive, but they may remain testing negative for SARS-CoV-2 RNA throughout the disease course when all testing agents work normally. In Wuhan where SARS-CoV-2 was initially identified, on February 12, ~15,000 incident cases were reported, far more than the numbers reported on the other dates which hardly exceeded 3,000. The inclusion of the clinically diagnosed cases with characteristic radiological findings regardless of the RNA testing result greatly contributed to the number, and COVID-19 was quickly controlled thereafter in Wuhan (3). In many other places in China, such cases have also been carefully managed in a similar way to laboratory-confirmed cases, which might have greatly contributed to the efficient control of COVID-19 (4).

However, the features of such “radiographically positive-only” cases have been rarely reported, and their epidemiological significance remains largely unknown. In this report, we comprehensively characterized such cases identified in multiple clusters from a large population-based prospective cohort in Feidong, Hefei, China, where careful and active case quarantine and contact tracing have been done, and compared them with confirmed COVID-19 cases and those with non-COVID-19 pneumonia. We further focused on a cluster where the transmission possibility of such cases were explored.

## Methods

### Cases

Since the identification of the first imported COVID-19 case in Hefei, China on January 23, 2020, a series of forceful control measures were immediately undertaken (5). We quickly formed an expert team comprising of epidemiologists and physicians and prospectively surveilled COVID-19 at the population level in Feidong, a county in Hefei with a population of ~1,081,000. This study was approved by the Ethics Committee of the Feidong People's Hospital, the eastern branch of First Affiliated Hospital of Anhui Medical University. Written informed consent was obtained from all participants. Reporting of the study conforms to broad EQUATOR guidelines (6).

Any individual with a recent (within 3 months) history of travel to Wuhan or other epidemic areas, or with relevant respiratory, digestive, and/or systemic symptoms and/or chest CT imaging suggesting COVID-19 before SARS-CoV-2 RNA assay, was considered suspicious and was immediately quarantined and closely monitored (7). The close contacts of both the above suspected and confirmed COVID-19 cases were quickly and comprehensively traced after careful and detailed interview and field investigation. All suspected cases and the contacts immediately underwent SARS-CoV-2 RNA testing by rRT-PCR assays of respiratory (throat swab and sputum) specimens. COVID-19 case was confirmed by ≥2 positive rRT-PCR tests performed, separated by ≥24 h, based on the criteria by the WHO (8–10).

During the quarantine of those not yet confirmed to have COVID-19, any case with new-onset symptoms received CT scanning, and those with positive CT imaging findings were referred to hospital for further management, together with confirmed cases. All hospitalized cases underwent sequential SARS-CoV-2 RNA assays: Virus detection was repeated 1 day after the first positive and the first negative tests for confirmation of the previous tests and every 3 days in other situations, until the discharge criteria were met. For those with positive CT findings but negative RNA tests throughout the disease course, their blood, urine, and feces samples were additionally tested for SARS-CoV-2 RNA (each ≥2 times). For all hospitalized cases, detection of other common respiratory pathogens was also performed using rRT-PCR assays of extracted nucleic acid samples from respiratory specimens, indirect immunofluorescence assay of the corresponding IgM antibodies in blood specimens, and/or pathogen culture as detailed previously (7).

Four authors (XWZ, LLZ, JJX, and CYZ) directly cared for the hospitalized cases. The management of suspected and confirmed COVID-19 cases followed the WHO (11) and the National Health Commission of China (4), where timely oxygen supplement had been recommended for COVID-19 cases regardless of severity of disease.

Fitness for discharge was based on abatement of fever for ≥3 days and resolved respiratory and other major relevant symptoms and signs, with substantial improvement of chest radiographic evidence (for all hospitalized cases) and viral clearance in respiratory samples as demonstrated by ≥2 consecutively negative rRT-PCR tests for SARS-CoV-2 separated by ≥24 h (additionally required for laboratory-confirmed cases) (12). Discharged cases continued to be closely monitored and isolated for ≥2 weeks, followed by reexamination to exclude the relapse of infection.

We first included two groups of cases: Patients in Group 1 were confirmed to have COVID-19 and excluded from other pathogen infections; individuals in Group 2 had clear positive exposure history, COVID-19-associated symptoms [majorly fever and/or cough (2, 7)], and typical CT findings for COVID-19, but they tested negative for SARS-CoV-2 RNA (≥3 tests) and other respiratory pathogens throughout the disease course. Three cases testing negative for SARS-CoV-2 at the first sampling but positive in the following ones were categorized into Group 1 of laboratory-confirmed COVID-19 cases; the initial negative tests in these cases could be due to false-negative findings. People in Groups 1 and 2 were from eight clusters. We further detailed a cluster to explore the epidemiological significances of cases in Group 2. We further included patients with other non-COVID-19 pneumonia as Group 3, who did not have an exposure history and tested negative for SARS-CoV-2 RNA throughout the disease course (≥3 tests), and who had symptoms and positive CT findings suggesting pneumonia; through pathogen analyses, among 62 patients in Group 3, 6 (10%) had non-COVID-19 viral pneumonia, 47 (76%) bacterial pneumonia, and 9 (15%) mycoplasma pneumonia.

All included patients underwent CT scan, and radiological diagnosis was based on CT scan findings in our study. Typical CT scan findings for COVID-19 (e.g., multifocal mottling, patchy shadows, ground-glass opacities) were in accordance with the guidelines of the WHO (1) and the National Health Commission of China (4), and have been shown and described in our previous publication (7). We prospectively collected information on demographic, baseline, clinical, radiological, and laboratory (blood routine, coagulation function, blood chemistry, and infection-associated biomarkers) features, management, and outcomes and recorded them in a standardized database.

### Tests

Respiratory tract (throat swabs and sputum) samples were immediately collected from all cases at admission and contacts by otolaryngologists after suspicion of disease and again after 24 h, and then sent to the designated authoritative laboratories of Hefei City and/or Anhui Provincial Centers for Disease Control and Prevention (CDCs) to be tested for SARS-CoV-2 using rRT-PCR. Repeated tests for SARS-CoV-2 were performed in patients ascertained to have COVID-19 to show viral clearance before discharge from hospital or discontinuation of isolation. Cases in Groups 2 (throughout the disease course) and 3 underwent SARS-CoV-2 RNA tests for respiratory samples for at least three times. Sample collection, processing, and laboratory diagnostic-testing followed the WHO recommendations (8, 13, 14) and the CDC guidelines (15, 16). Detection of SARS-CoV-2 and tests to rule out infection from other common potentially causative respiratory pathogens which were performed in all patients are detailed as follows.

Throat swab specimens were collected with synthetic fiber swabs, each of which was placed into a separate sterile collection tube containing viral preservation and transport medium. Viral RNA was extracted (17) and tested using rRT-PCR with SARS-CoV-2-specific primers and probes (18, 19) using the China CDC recommended kit (BioGerm, Shanghai, China). The diagnostic assay has three nucleocapsid gene targets and one positive control target (20). If two targets (open reading frame 1a or 1b, nucleocapsid protein) tested positive on specific rRT-PCR, the case would be considered to be laboratory confirmed. A cycle threshold (Ct) value <37 was defined as a positive test, and a Ct value of ≥40 was defined as a negative test. A medium load, defined as a Ct value of 37 to <40, required confirmation by retesting. If the repeated Ct value was <40 and an obvious peak was observed, or if the repeated Ct value was <37, the retest was deemed positive (13, 17). These diagnostic criteria were based on the recommendation by the China National Institute for Viral Disease Control and Prevention (21). For the open reading frame 1ab target, the forward primer sequence was CCCTGTGGGTTTTACACTTAA, the reverse primer sequence ACGATTGTGCATCAGCTGA, and the probe sequence 5′-FAM-CCGTCTGCGGTATGTGGAAAGGTTATGG-BHQ1-3′; for the nucleoprotein target, the forward primer sequence was GGGGAACTTCTCCTGCTAGAAT, the reverse primer sequence CAGACATTTTGCTCTCAAGCTG, and the probe sequence 5′-FAM-TTGCTGCTGCTTGACAGATT-TAMRA-3′. The detection limit of the rRT-PCR assay was assumed to be 200 copies/ml. The sensitivity and specificity for ≥3 tests were estimated to be >97% and about 100%, respectively (22).

Detection of common viral respiratory pathogens including influenza A (H1N1, H1-2009, H3N2, and N7N9) and B, parainfluenza (types 1–4), respiratory syncytial virus, human rhinovirus or enterovirus, adenovirus, and common coronavirus strains known to cause illness in humans (HCoV-229E, HCoV-Nl63, HCoV-Oc43, HCoV-HKU1, SARS-CoV, and MERS-CoV); bacterial pathogens including *Bordetella pertussis, Bordetella parapertussis, Chlamydophila pneumoniae, Mycoplasma pneumoniae*; and methicillin-resistant *Staphylococcus aureus* was performed using rRT-PCR assays of extracted nucleic acid samples from respiratory specimens (23, 24). The IgM antibodies of nine respiratory pathogens including *Legionella pneumophila, mycoplasma pneumoniae, Q fever rickettsia, Chlamydia pneumoniae*, adenovirus, respiratory syncytial virus, influenza A and B, and parainfluenza in blood specimens were also examined using the indirect immunofluorescence assay. Routine fungal examinations were also performed. Bacteria and fungi culture were done after admission.

### Statistics

Categorical data were presented as count (percentage), and continuous data as median (interquartile range). We compared data across groups using χ^2^ or Fisher's exact test where appropriate for categorical data, and Kruskal–Wallis test for continuous data. When a significant difference was detected, *post-hoc* multiple comparisons were performed. Temporal changes of probability of hospitalization were illustrated using the Kaplan–Meier method and compared across groups using the log-rank test. Analyses were performed using the R 3.6.2 software (https://www.r-project.org/), and a two-sided *p* value < 0.05 suggested statistical significance.

## Results

Through careful case quarantine and active contact tracing, a total of 14,839 people underwent SARS-CoV-2 RNA testing, and 36, 27, and 62 cases were included into Groups 1, 2, and 3, respectively. All cases in Group 2 tested negative for SARS-CoV-2 RNA in respiratory and other samples. Demographic and baseline characteristics including sex, age, smoking status, and coexisting chronic conditions were all similar across the three groups (Table 1). On admission, fever was more common in Group 2 (96%) than in Groups 1 (75%) and 3 (71%). Median interval from illness onset to expectoration among patients was longer in Group 1 (2 d) than in Group 3 (0 d) in people with wet cough. Headache (17 vs. 2%) and chest discomfort (19 vs. 3%) was more frequent in Group 1 than in Group 3. Diarrhea or change of stool character occurred more often in Group 1 (39%) than in Groups 2 (15%) and 3 (6%). Median oxygen saturation recorded in room air was slightly lower in Group 1 (97%) than in Group 3 (98%). All the other symptoms, signs, and radiological features were comparable across groups.

**Table 1:** Demographic, baseline, clinical, and radiological features.

**Characteristics**	**Group 1 *(n = 36)***	**Group 2 (*n* = 27)**	**Group 3 (*n* = 62)**	***p***
**Demographic & baseline**				
Male sex	18 (50)	15 (56)	35 (56)	0.818
Age (y)	42 (24–57)	41 (31–56)	49 (35–65)	0.221
Current smoker	2 (6)	0 (0)	4 (6)	0.479
Chronic obstructive pulmonary disease	1 (3)	0 (0)	1 (2)	1.000
Other respiratory system diseases	0 (0)	0 (0)	2 (3)	0.712
Hypertension	7 (19)	2 (7)	12 (19)	0.337
Coronary heart disease	0 (0)	0 (0)	2 (3)	0.712
Other heart diseases	0 (0)	0 (0)	1 (2)	1.000
Diabetes	4 (11)	0 (0)	3 (5)	0.197
Liver disease	0 (0)	1 (4)	0 (0)	0.216
Other digestive system diseases	1 (3)	0 (0)	2 (3)	1.000
Brain disease	0 (0)	0 (0)	2 (3)	0.712
Other nervous system diseases	0 (0)	0 (0)	1 (2)	1.000
Other chronic conditions	2 (6)	0 (0)	0 (0)	0.127
**Clinical & radiological features on admission**				
Fever	27 (75)	26 (96)	44 (71)	**0.028**
Intermittent fever	8/27 (30)	7/26 (27)	11/44 (25)	0.913
Body temperature on admission (°C)	37.1 (36.6–38.0)	37.3 (36.8–38.0)	37.0 (36.6–38.2)	0.803
Highest body temperature (°C)	38.5 (38.3–39.0)	38.2 (37.8–38.5)	38.5 (38.0–38.8)	0.166
Days from illness onset to fever	0 (0–1)	0 (0–4)	0 (0–0)	***0.063***
Days from illness onset to highest fever	3 (0–6)	2 (0–7)	2 (0–6)	0.922
Duration of fever (d)	4 (3–7)	3 (2–6)	5 (3–8)	0.488
Chill	7 (19)	5 (19)	6 (10)	0.327
Cough	31 (86)	23 (85)	58 (94)	0.296
Days from illness onset to cough	0 (0–2)	0 (0–2)	0 (0–0)	0.783
Expectoration	20 (56)	19 (70)	36 (58)	0.449
Days from illness onset to expectoration	2 (0–5)	0 (0–4)	0 (0–0)	**0.025**
Hemoptysis	1 (3)	0 (0)	2 (3)	1.000
Myalgia	0 (0)	1 (4)	0 (0)	0.216
Fatigue	0 (0)	2 (7)	1 (2)	0.194
Eye discomfort	0 (0)	0 (0)	1 (2)	1.000
Headache	6 (17)	2 (7)	1 (2)	**0.013**
Dizziness	4 (11)	2 (7)	1 (2)	***0.084***
Chest discomfort	7 (19)	3 (11)	2 (3)	**0.022**
Chest pain	2 (6)	1 (4)	0 (0)	0.125
Shortness of breath	0 (0)	0 (0)	2 (3)	0.712
Backache	1 (3)	0 (0)	0 (0)	0.504
Rhinorrhea	2 (6)	0 (0)	4 (6)	0.479
Nasal congestion	4 (11)	0 (0)	5 (8)	0.229
Sore throat	2 (6)	2 (7)	5 (8)	1.000
Sneezing	3 (8)	0 (0)	1 (2)	0.126
Diarrhea or change of stool character	14 (39)	4 (15)	4 (6)	** <0.001**
Anorexia	0 (0)	1 (4)	1 (2)	0.468
Nausea	6 (17)	2 (7)	4 (6)	0.310
Vomiting	2 (6)	2 (7)	2 (3)	0.645
Abdominal discomfort	2 (6)	2 (7)	6 (10)	0.909
Oxygen saturation (%) in room air	97 (97–98)	98 (97–98)	98 (98–98)	**0.003**
Respiratory rate (breaths/min)	20 (20–21)	20 (20–21)	20 (20–21)	0.631
Pulse (beats/min)	87 (78–98)	92 (88–110)	88 (80–106)	0.116
Systolic blood pressure (mmHg)	120 (120–137)	130 (120–132)	132 (121–145)	***0.082***
Diastolic blood pressure (mmHg)	78 (70–88)	83 (76–89)	81 (74–88)	0.209
Days from illness onset to first CT scan	3 (2–6)	3 (1–7)	3 (1–6)	0.902
Days from illness onset to CT pneumonia	4 (2–7)	3 (1–7)	3 (2–7)	0.620
Bilateral disease on CT scan	21 (58)	13 (48)	28 (45)	0.447

*Patients in Group 1 were confirmed to have COVID-19 and excluded from other pathogen infections; individuals in Group 2 had clear exposure history, symptoms, and typical CT findings for COVID-19, but they tested negative for SARS-CoV-2 RNA and other respiratory pathogens throughout the disease course; we further included patients with other non-COVID-19 pneumonia as Group 3, who did not have an exposure history and tested negative for SARS-CoV-2 RNA throughout the disease course, and who had symptoms and positive CT findings suggesting pneumonia. *p* values < 0.05 are shown in bold, and *p* values ≥ 0.05 and < 0.10 are shown in both bold and italic*

Leucocyte count on admission was greater in Group 3 (median, 6.22 × 10^9^/L) than in Groups 1 (5.01 × 10^9^/L) and 2 (5.09 × 10^9^/L), and it more often decreased in Group 1 (33%) than in Group 3 (10%) (Table 2). Neutrophil count was also larger in Group 3 (3.95 × 10^9^/L) than in Groups 1 (3.00 × 10^9^/L) and 2 (3.00 × 10^9^/L), and it more often decreased in Group 1 (25%) than in Groups 2 (11%) and 3 (5%). Monocyte count was greater in Group 3 (0.56 × 10^9^/L) than in Groups 1 (0.43 × 10^9^/L) and 2 (0.46 × 10^9^/L), and it increased more often in Group 3 (26%) than in Group 1 (0%). Eosinophil count was also larger in Group 3 (0.04 × 10^9^/L) than in Groups 1 and 2 (both 0.01 × 10^9^/L), and it decreased more often in Group 2 (59%) than in Group 3 (31%). Prothrombin time was slightly longer in Group 3 (11.7 s) than in Group 1 (11.2 s), and accordingly international normalized ratio was larger in Group 3 (1.01) than in Group 1 (0.96). D-dimer level was lower in Group 1 (0.27 μg/L) than in Groups 2 (0.44 μg/L) and 3 (0.43 μg/L). Total protein level was lower in Group 3 (67.4 g/L) than in Groups 1 (68.7 g/L) and 2 (70.2 g/L), and it decreased more often in Group 3 (31%) than Group in 1 (8%). Albumin level was slightly lower in Group 3 (41.3 g/L) than in Groups 1 (42.8 g/L) and 2 (43.8 g/L), and it decreased more often in Group 3 (40%) than in Group 1 (14%). Sodium level was slightly lower in Group 3 (139.2 mmol/L) than in Group 1 (140.4 mmol/L). C-reactive protein level was higher in Group 3 (27.10 mg/L) than in Groups 1 (8.31 mg/L) and 2 (7.94 mg/L), and erythrocyte sedimentation rate was faster in Group 3 (48 mm/h) than in Groups 1 (33 mm/h) and 2 (29 mm/h). Procalcitonin level increased less often in Group 1 (19%) than in Groups 2 (44%) and 3 (60%).

**Table 2:** Laboratory features on admission.

**Characteristics**	**Group 1 (*n* = 36)**	**Group 2 (*n* = 27)**	**Group 3 (*n* = 62)**	**p_**123**_**
**Blood routine**				
White blood cell count (× 10^9^/L)	5.01 (3.65–6.62)	5.09 (4.00–6.25)	6.22 (4.76–8.01)	**0.004**
Increased	0 (0)	4 (15)	5 (8)	**0.007**
Decreased	12 (33)	6 (22)	6 (10)	
Neutrophil count (× 10^9^/L)	3.00 (1.90–4.10)	3.00 (2.30–4.60)	3.95 (3.10–5.70)	**0.007**
Increased	0 (0)	3 (11)	5 (8)	**0.013**
Decreased	9 (25)	3 (11)	3 (5)	
Lymphocyte count (× 10^9^/L)	1.27 (0.94–1.69)	1.23 (0.95–1.40)	1.32 (0.95–1.59)	0.708
Increased	1 (3)	0 (0)	0 (0)	0.261
Decreased	7 (19)	6 (22)	7 (11)	
Monocyte count (× 10^9^/L)	0.43 (0.36–0.55)	0.46 (0.37–0.67)	0.56 (0.49–0.83)	**0.001**
Increased	0 (0)	3 (11)	16 (26)	**0.002**
Eosinophil count (× 10^9^/L)	0.01 (0.00–0.06)	0.01 (0.00–0.04)	0.04 (0.01–0.12)	** <0.001**
Increased	0 (0)	0 (0)	3 (5)	**0.048**
Decreased	18 (50)	16 (59)	19 (31)	
Basophil count (× 10^9^/L)	0.01 (0.01–0.03)	0.01 (0.01–0.02)	0.02 (0.01–0.03)	***0.088***
Red blood cell (× 10^12^/L)	4.58 (4.29–4.91)	4.67 (4.11–5.14)	4.41 (4.03–4.87)	0.134
Increased	4 (11)	1 (4)	5 (8)	0.710
Decreased	0 (0)	1 (4)	2 (3)	
Hemoglobin (g/L)	137 (128–152)	136 (124–147)	129 (119–143)	***0.068***
Increased	5 (14)	2 (7)	4 (6)	0.669
Decreased	2 (6)	1 (4)	6 (10)	
Hematocrit (%)	42.1 (38.4–46.1)	40.6 (37.0–45.1)	39.5 (36.3–42.8)	***0.051***
Increased	0 (0)	1 (4)	2 (3)	0.277
Decreased	4 (11)	5 (19)	16 (26)	
Platelet count (× 10^9^/L)	166 (118–209)	161 (116–191)	166 (132–220)	0.389
Increased	1 (3)	0 (0)	2 (3)	0.841
Decreased	5 (14)	3 (11)	5 (8)	
**Coagulation function**				
Prothrombin time (s)	11.2 (10.6–11.7)	11.4 (10.6–12.1)	11.7 (11.0–12.7)	**0.039**
Increased	0 (0)	0 (0)	0 (0)	0.824
Decreased	6 (17)	3 (11)	9 (15)	
International normalized ratio	0.96 (0.91–1.01)	0.98 (0.91–1.04)	1.01 (0.95–1.10)	**0.047**
Increased	0 (0)	1 (4)	2 (3)	0.612
Decreased	2 (6)	1 (4)	1 (2)	
Activated partial thrombin time (s)	26.9 (24.5–29.2)	28.0 (25.5–29.5)	28.7 (25.1–32.7)	0.155
Increased	1 (3)	1 (4)	1 (2)	0.391
Decreased	0 (0)	1 (4)	0 (0)	
Fibrinogen (g/L)	2.95 (2.59–3.69)	2.95 (2.78–3.49)	3.15 (2.87–4.05)	0.289
Increased	5 (14)	5 (19)	16 (26)	0.143
Decreased	2 (6)	2 (7)	0 (0)	
D–dimer (μg/L)	0.27 (0.19–0.43)	0.44 (0.27–0.57)	0.43 (0.32–0.50)	**0.031**
Increased	2 (6)	3 (11)	8 (13)	0.321
Decreased	0 (0)	1 (4)	0 (0)	
**Blood chemistry**				
Total protein (g/L)	68.7 (66.4–73.7)	70.2 (67.8–72.6)	67.4 (62.9–70.0)	**0.009**
Decreased	3 (8)	4 (15)	19 (31)	**0.022**
Albumin (g/L)	42.8 (41.8–45.1)	43.8 (40.2–45.1)	41.3 (38.1–43.9)	**0.004**
Decreased	5 (14)	6 (22)	25 (40)	**0.014**
Pre-albumin (mg/L)	164 (146–216)	165 (131–214)	151 (126–203)	0.366
Decreased	22 (61)	19 (70)	41 (66)	0.740
ALT (U/L)	25 (14–37)	22 (14–39)	20 (14–36)	0.816
Increased	5 (14)	4 (15)	9 (15)	0.994
AST (U/L)	25 (23–34)	26 (19–41)	23 (18–34)	0.254
Increased	6 (17)	7 (26)	11 (18)	0.820
Decreased	0 (0)	0 (0)	1 (2)	
AKP (U/L)	78 (69–88)	66 (57–88)	72 (62–95)	0.252
Increased	0 (0)	1 (4)	3 (5)	0.438
Total bilirubin (μmol/L)	11.8 (9.8–16.7)	14.6 (9.7–18.5)	13.8 (10.7–17.7)	0.692
Increased	4 (11)	4 (15)	7 (11)	0.876
Direct bilirubin (μmol/L)	2.7 (2.1–3.5)	3.2 (2.0–4.0)	2.8 (2.1–3.7)	0.839
Increased	0 (0)	0 (0)	1 (2)	1.000
Serum creatinine (μmol/L)	65.7 (52.3–73.4)	64.5 (57.5–78.5)	66.1 (52.9–76.1)	0.823
Increased	0 (0)	0 (0)	1 (2)	0.908
Decreased	3 (8)	1 (4)	5 (8)	
Blood urea nitrogen (mmol/L)	3.89 (3.33–4.99)	3.51 (3.15–4.59)	3.79 (3.24–4.50)	0.269
Increased	1 (3)	0 (0)	2 (3)	0.287
Decreased	2 (6)	5 (19)	12 (19)	
Creatine kinase (U/L)	67 (53–104)	83 (60–122)	80 (58–104)	0.531
Increased	2 (6)	2 (7)	4 (6)	1.000
Creatine kinase type-MB (U/L)	11 (9–13)	10 (8–12)	9 (8–11)	***0.055***
Increased	0 (0)	0 (0)	1 (2)	0.708
Decreased	1 (3)	0 (0)	4 (6)	
Lactate dehydrogenase (U/L)	205 (166–234)	183 (161–211)	180 (157–212)	0.494
Increased	6 (17)	3 (11)	8 (13)	0.836
Glucose (mmol/L)	4.87 (4.41–5.00)	5.03 (4.62–5.57)	4.93 (4.58–5.48)	0.405
Increased	0 (0)	2 (7)	8 (13)	***0.082***
Decreased	0 (0)	0 (0)	1 (2)	
Potassium (mmol/L)	4.09 (3.75–4.41)	4.10 (3.80–4.30)	4.16 (3.94–4.44)	0.361
Decreased	3 (8)	1 (4)	4 (6)	0.802
Sodium (mmol/L)	140.4 (139.4–141.7)	140.6 (138.5–141.8)	139.2 (137.7–140.8)	**0.011**
Decreased	1 (3)	1 (4)	7 (11)	0.342
Chloride (mmol/L)	102.7 (101.4–104.9)	103.3 (100.6–105.0)	102.1 (100.0–105.0)	0.305
Increased	1 (3)	0 (0)	0 (0)	0.534
Decreased	1 (3)	0 (0)	3 (5)	
Calcium (mmol/L)	2.27 (2.19–2.34)	2.28 (2.22–2.36)	2.23 (2.16–2.33)	0.282
Decreased	5 (14)	4 (15)	12 (19)	0.747
**Infection–associated biomarkers**				
C-reactive protein (mg/L)	8.31 (5.69–23.90)	7.94 (5.10–28.50)	27.10 (7.19–66.28)	**0.006**
Increased	19 (53)	14 (52)	40 (65)	0.387
Procalcitonin (ng/ml)	0.043 (0.031–0.090)	0.044 (0.032–0.067)	0.059 (0.031–0.091)	0.311
Increased	7 (19)	12 (44)	37 (60)	**0.001**
Erythrocyte sedimentation rate (mm/h)	33 (20–51)	29 (20–51)	48 (26–80)	**0.034**
Increased	23 (64)	14 (52)	42 (68)	0.358

*Patients in Group 1 were confirmed to have COVID-19 and excluded from other pathogen infections; individuals in Group 2 had clear exposure history, symptoms, and typical CT findings for COVID-19, but they tested negative for SARS-CoV-2 RNA and other respiratory pathogens throughout the disease course; we further included patients with other non-COVID-19 pneumonia as Group 3, who did not have an exposure history and tested negative for SARS-CoV-2 RNA throughout the disease course, and who had symptoms and positive CT findings suggesting pneumonia. *p* values < 0.05 are shown in bold, and *p* values ≥ 0.05 and < 0.10 are shown in both bold and italic*.

**Figure 1:**
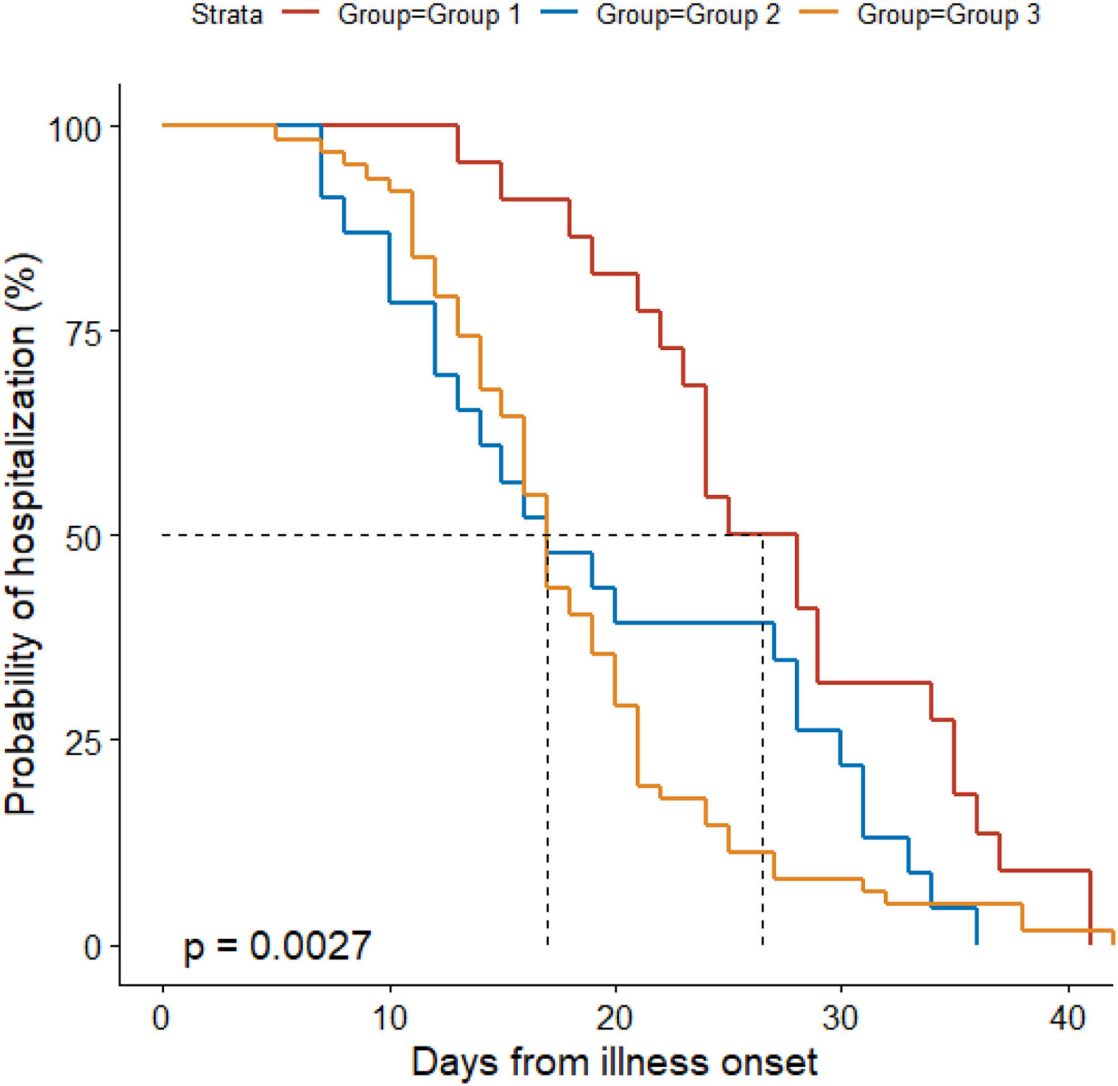
Probability of hospitalization by duration from illness onset in the three groups. Patients in Group 1 were confirmed to have COVID-19 and excluded from other pathogen infections; individuals in Group 2 had clear exposure history, symptoms, and typical CT findings for COVID-19, but they tested negative for SARS-CoV-2 RNA and other respiratory pathogens throughout the disease course; we further included patients with other non-COVID-19 pneumonia as Group 3, who did not have an exposure history and tested negative for SARS-CoV-2 RNA throughout the disease course, and who had symptoms and positive CT findings suggesting pneumonia.

Antibacterial therapy was less often used in Group 1 (86%) than in Group 3 (100%) (Table 3). Among people receiving antibacterial therapy, combination therapy was more often used in Group 3 (66%) than in Groups 1 (19%) and 2 (35%). Levofloxacin was more frequently administered in Group 3 (89%) than in Groups 1 (58%) and 2 (67%). Group 3 (23%) received less often lopinavir/ritonavir than Groups 1 (72%) and 2 (48%). Ribavirin was more often used in Group 3 (90%) than in Group 1 (56%). Group 1 (17%) received more often corticosteroids than Groups 2 (0%) and 3 (2%). Group 2 (63%) received less often oxygen therapy than Groups 1 (97%) and 3 (87%), and it (63%) used less frequently nasal cannula than Groups 1 (89%) and 3 (87%). Non-invasive mechanical ventilation was only used in Group 1 (8%). Median time from illness onset to discharge was longer in Group 1 (27 d) than in the other groups (both 17 d) (Figure 1).

**Table 3:** Management and outcomes.

**Characteristics**	**Group 1 (*n* = 36)**	**Group 2 (*n* = 27)**	**Group 3 (*n* = 62)**	**p_**123**_**
Days from illness onset to first hospitalization	3 (2–6)	3 (1–7)	3 (2–7)	0.540
Days from illness onset to blood sampling	4 (2–6)	4 (2–9)	4 (3–7)	0.831
Days from illness onset to first respiratory sampling	6 (2–7)	7 (2–11)	5 (4–9)	0.426
Antibacterial therapy	31 (86)	26 (96)	62 (100)	**0.004**
Days from illness onset to antibacterial therapy	4 (2–6)	5 (2–10)	3 (2–7)	0.771
Combination antibacterial therapy	6/31 (19)	9/26 (35)	41/62 (66)	** <0.001**
Levofloxacin	21 (58)	18 (67)	55 (89)	**0.002**
Moxifloxacin	10 (28)	8 (30)	22 (35)	0.701
Other antibacterial therapy	6 (17)	11 (41)	31 (50)	**0.005**
Antiviral therapy	36 (100)	27 (100)	61 (98)	1.000
Days from illness onset to antiviral therapy	4 (2–6)	4 (2–9)	4 (2–7)	0.696
Lopinavir/ritonavir	26 (72)	13 (48)	14 (23)	** <0.001**
Interferon	36 (100)	24 (89)	57 (92)	0.109
Ribavirin	20 (56)	21 (78)	56 (90)	** <0.001**
Osetamir	10 (28)	8 (30)	7 (11)	***0.053***
Arbidol	10 (28)	10 (37)	15 (24)	0.463
Other antiviral therapy	12 (33)	19 (70)	54 (87)	** <0.001**
Corticosteroids	6 (17)	0 (0)	1 (2)	**0.003**
Oxygen therapy	35 (97)	17 (63)	54 (87)	**0.001**
Nasal cannula	32 (89)	17 (63)	54 (87)	**0.011**
Days from illness onset to nasal cannula use	4 (2–7)	3 (2–6)	4 (3–7)	0.764
Noninvasive mechanical ventilation	3 (8)	0 (0)	0 (0)	**0.032**
Days from illness onset to discharge	27 (22–35)	17 (12–30)	17 (13–21)	** <0.001**

*Patients in Group 1 were confirmed to have COVID-19 and excluded from other pathogen infections; individuals in Group 2 had clear exposure history, symptoms, and typical CT findings for COVID-19, but they tested negative for SARS-CoV-2 RNA and other respiratory pathogens throughout the disease course; we further included patients with other non-COVID-19 pneumonia as Group 3, who did not have an exposure history and tested negative for SARS-CoV-2 RNA throughout the disease course, and who had symptoms and positive CT findings suggesting pneumonia. *p* values < 0.05 are shown in bold, and *p* values ≥ 0.05 and < 0.10 are shown in both bold and italic*.

**Figure 2:**
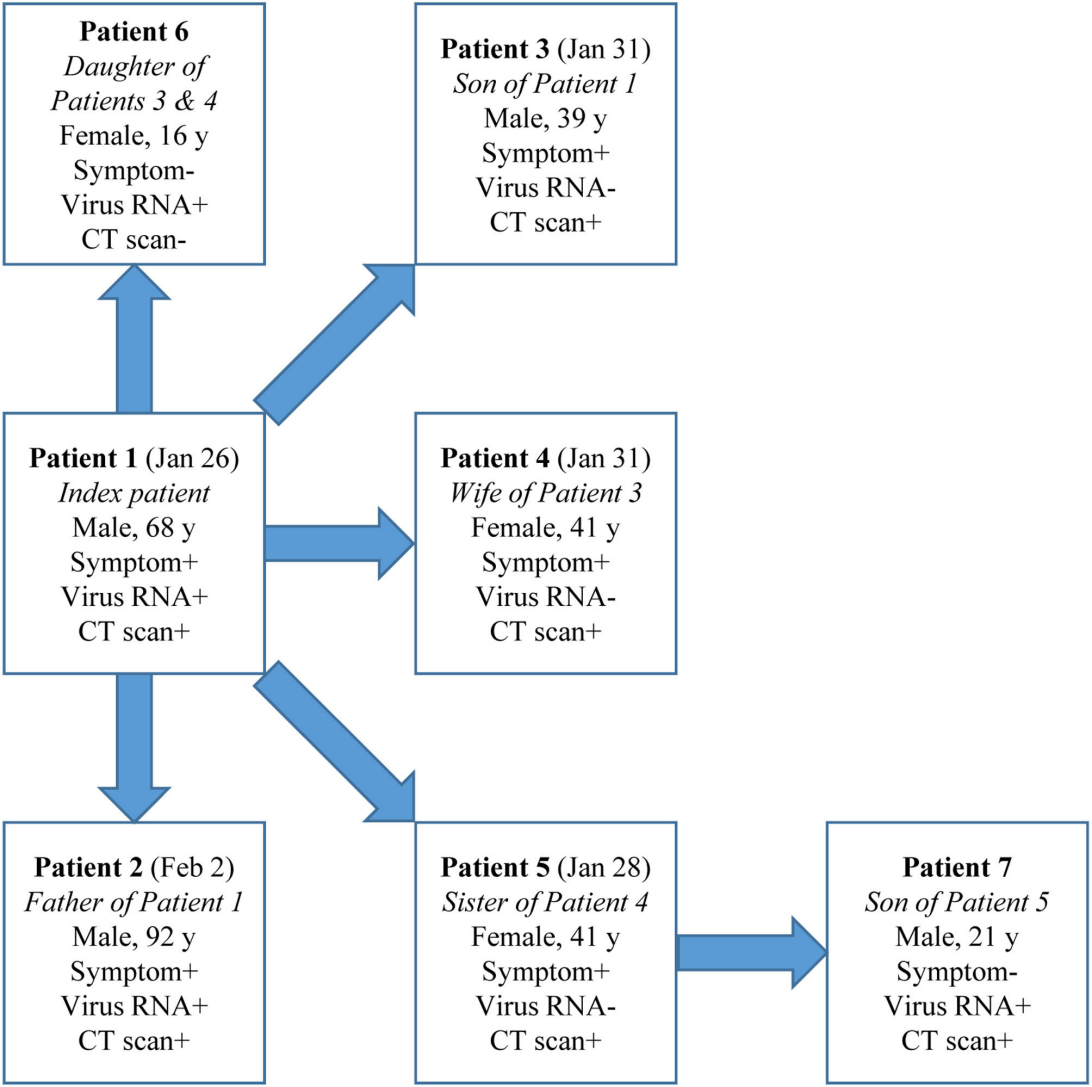
A COVID-19 cluster. Dates are for symptom onset.

Cases in Group 2 could be a source of infection as demonstrated in a cluster (Figure 2). Patient Index, a 68-year-old male, had illness onset on Jan 26 and tested positive for SARS-CoV-2 RNA and had positive CT findings. After his exposure he contacted five other people without exposure to Wuhan or other epidemic centers or contact with suspected or confirmed COVID-19 cases: Patient 2, his 92-year-old father, during the daily care, and Patients 3–6 during a family get-together. Interestingly, Patients 3–5 (39–41 years) all had symptoms and positive CT findings but tested negative for SARS-CoV-2 RNA throughout the disease course. However, Patient 6, a 16-year-old female, tested positive for SARS-CoV-2 RNA but had neither symptoms nor positive CT findings. Patient 7, the son of Patient 5, a radiographically positive-only case, later tested positive for SARS-CoV-2 RNA during contact quarantine and had positive CT findings, but remained asymptomatic during the disease course. Except for Patient 5, Patient 7 did not have any other possible exposure histories.

## Discussion

This report comprehensively characterized the symptomatic contacts of confirmed COVID-19 cases who had positive radiological presentations but remained testing negative for SARS-CoV-2 RNA and all the other common respiratory pathogens from first respiratory sampling through discharge, which represented a relatively large proportion compared to their counterparts of confirmed cases. We further compared them with confirmed cases and non-COVID-19 pneumonia cases in a head-to-head manner, and described a disease cluster where such cases appeared to be contagious. Our findings add important information on COVID-19 control to literature.

These radiographically positive-only patients kept testing negative for SARS-CoV-2 RNA possibly due to the following reasons: first, the virus appeared transiently in examined samples since illness onset but before first sampling, or even during the incubation period, when strong infectivity could be already present (7); the cases might be majorly infective during these periods. Afterwards, all samples remained testing negative for SARS-CoV-2 RNA throughout the disease course. The median duration from illness onset to first respiratory sampling was 7 days for these patients, which was similar to those for the other groups. If this most likely explanation makes sense, in addition to the incubation period, there could be a high risk that these cases transmitted COVID-19 to others before being quarantined, which markedly increases the complexity and difficulty of disease control. This possibility highlights the importance of early, careful, and comprehensive quarantine and monitoring of all people with any epidemiological history. Second, the sensitivity of the RNA testing might not be high enough. The testing agents in our study were uniformly used and testing was centrally performed in the designated CDC where authoritative international guidelines were carefully followed (8–10). Respiratory tract samples were collected by otolaryngologists with expertise in such sampling, to ensure that samples were properly collected. Besides swabs, sputum samples were also collected and tested. Sample collection, processing, and laboratory diagnostic testing followed the WHO recommendations and the CDC guidelines. Tests were repeated with short intervals until discharge. Cases with negative SARS-CoV-2 RNA findings underwent at least three times of rRT-PCR tests, and parameters on the test detection limit (200 copies/ml) and sensitivity (>97% for ≥3 tests) made the explanations of low sample viral load or low test sensitivity for the negative SARS-CoV-2 RNA findings in Group 2 or 3 patients less likely (22). Furthermore, testing of all the other specimens all revealed negative results. It is less likely that the testing itself resulted in such a proportion of radiographically positive-only cases. Third, besides symptomatic confirmed cases and asymptomatic carriers, such cases may represent a novel COVID-19 infection subpopulation. While infected with SARS-CoV-2, the virus was hardly detectable in various specimens. They presented clinical, radiological, and laboratory features mostly similar to confirmed COVID-19 cases, and should be managed in the same way as confirmed cases. Of course, there could be coexistence of both the first and third possibilities.

**Figure 3:**
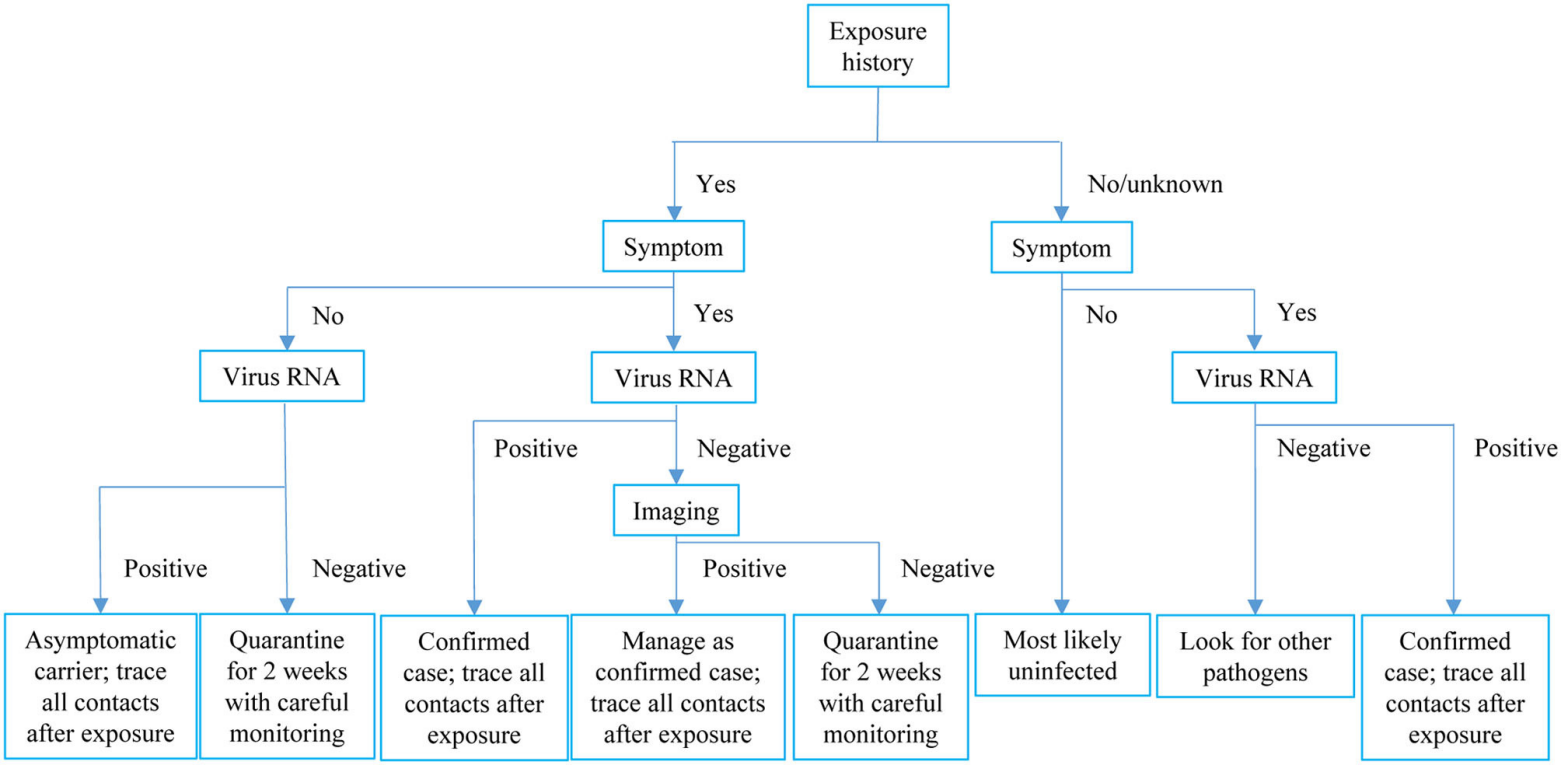
Recommended clinical pathway for COVID-19 in the resource-limited pandemic era.

In the cluster specially looked into, four types of COVID-19-relevant cases with positive epidemiological histories were observed: Symptomatic patients with both positive SARS-CoV-2 RNA and radiological findings; symptomatic cases with positive imaging findings but testing negative for virus RNA; asymptomatic patients with both positive virus RNA and radiological findings; and asymptomatic cases with positive virus RNA only but without any positive radiological findings. We found evidence that a radiologically positive-only case transmitted COVID-19 to another asymptomatic case, who most likely belonged to the first circumstance as discussed above. The diverse presentations of contacts of COVID-19 cases highlight the complexity of disease and the importance of comprehensive consideration of exposure history, symptom, virus RNA test, and radiological evidence when trying to exclude a case from quarantine. In this resource-limited pandemic era, we would like to recommend the management pathway shown in Figure 3.

Nearly all (96%) of such radiographically-positive-only patients presented with fever, which might suggest a more active and potent response to SARS-CoV-2 in them, potentially helping to eliminate the virus early or keep the virus load at a low level. However, they had less often diarrhea compared to confirmed cases. The laboratory presentation in Group 2 is suggestive - yet not conclusive - for SARS-CoV-2 infection. Leucocyte especially neutrophil, monocyte, and eosinophil counts were lower in such cases than non-COVID-19 cases, indicating the difference in anti-pathogen immune response. However, in such cases neutrophil did not decrease as often as in confirmed cases. D-dimer level was higher in such cases than confirmed ones. Compared to non-COVID-19 pneumonia patients, such cases had better nutrition status with higher total protein and albumin levels but markedly lower C-reactive protein level and slower erythrocyte sedimentation rate, suggesting that those patients had pathogen and systemic response different from other pneumonia cases. However, procalcitonin level more frequently increased in such patients compared to confirmed cases. While being more similar to COVID-19 cases, at least the severity of disease could be different in such cases, possibly due to the discrepant constitution.

Considering the more likely viral nature of infection in such cases, empirically combination antibacterial therapy and levofloxacin were less often administered in those cases than other pneumonia patients, while lopinavir/ritonavir was more frequently used. The disease being less severe, none of such patients received corticosteroids, and they received oxygen therapy (mostly through nasal cannula) much less frequently than both confirmed COVID-19 and other pneumonia cases.

Through active and careful management, the duration from illness onset to discharge was markedly shorter in such cases than confirmed cases, and similar to other pneumonia patients. All discharged patients in Groups 1 and 2 underwent another ≥3-week quarantine in a hotel or at home under careful surveillance, and no one had disease relapse. Reportedly, the median duration of SARS-CoV-2 shedding was 20 days, with the longest being 37 days (25). The total duration of quarantine in our study of on average at least 5 weeks could well-ensure that SARS-CoV-2 was eliminated or at least became undetectable in nearly all cases.

This report was first limited by the relatively small number of RNA- and/or imaging-positive cases. SARS-CoV-2 antibodies especially IgM were not examined, and the statuses of virus shedding and immunity response before the first sampling were unknown. The infection in Group 2 patients was strongly clinically indicated based on clear positive exposure history, and typical symptoms and CT findings for COVID-19. SARS-CoV-2 RNA test is the golden standard and the most commonly used method for diagnosing COVID-19 across guidelines and regulations, and in this study, all suspected cases and the contacts immediately underwent SARS-CoV-2 RNA testing by rRT-PCR assays of respiratory (throat swab and sputum) specimens, which are the most commonly recommended specimens across guidelines and regulations and which are more easily obtainable and more practicable. A Finland population-based study showed that the clinical sensitivity of SARS-CoV-2 RT-PCR testing was only moderate at best, with relatively high false-negative rates (26), and a single swab test might not be sufficient (27); the sensitivity was even lower among smokers (28). Test sensitivity increased with more test times, and were 72.4, 89.8, 97.3, and 100.0% for the first, second, third, and seventh tests, respectively (22). In this study, patients with negative SARS-CoV-2 findings underwent at least three times of rRT-PCR tests. Notably, it has been previously shown that throat swab specimens might have lower viral loads than nasopharyngeal samples (29), and that nasopharyngeal samples are best at sensitivity detection, especially in early stages of disease and in asymptomatic individuals (30). The non-invasive specimen saliva/sputum also exhibited high sensitivity (87%) and specificity (98%) for the detection of SARS-COV-2 (31), and had similar sensitivity for detecting SARS-CoV-2 with nasopharyngeal samples (32); however, in a pediatric population, the sensibility of the saliva test was not high enough to replace the use of swab for COVID-19 diagnosis (33). In this study, we used respiratory specimens of both throat swab and sputum. We were unable to do sampling of lower respiratory tract secretions; while further obtaining specimens for testing via bronchoalveolar lavage might have contributed to a more definitive diagnosis, most of the patients did not consent to undergo such a procedure considering the relatively invasive nature of this technique. Furthermore, bronchoalveolar lavage was not mandatorily required for such cases according to guidelines and regulations, and during the early phase of the pandemic, the bronchoscopy rooms in our institutions had been closed due to the nature of the disease being largely unknown then and for fear of disease spread. Antibody tests would help with more definitive diagnosis. Rapid antigen test had up to 98% sensitivity and 100% specificity for detecting COVID-19 in persons with mild symptoms (34, 35). However, most of the patients in Group 2 were identified at a relatively early phase of the pandemic (only 1 of the 27 cases was hospitalized after March 2020), when antibody tests, which would also not be considered the golden standard, had not been available or popular then. Notably, the reliability of serological tests may vary and requires critical validation (36, 37). For Group 2 patients, besides common respiratory (throat swab and sputum) specimens, we also examined their blood, urine, and feces samples, and all samples tested negative. Nevertheless, our study would at least suggest that SARS-CoV-2 RNA testing by rRT-PCR assays of common respiratory (throat swab and sputum) specimens, the widely accepted “golden standard” for diagnosing COVID-19, might be sometimes insufficient, and that further studies with some other further procedures (e.g., testing via bronchoalveolar lavage or specific antibodies) would be warranted for Group 2-like patients, namely, the SARS-CoV-2 RNA-negative (tested using common respiratory specimens), radiographically positive, symptomatic contacts of COVID-19 cases. This study would then be considered more as a hypothesis-generating study.

The strengths of this report lie in the prospective population-based cohort design, analyses of multiple clusters, identification of a novel COVID-19 infection subtype, repeated testing of multiple samples, and proposal of a new practical clinical management pathway.

In conclusion, among close contacts of confirmed COVID-19 cases, some may present with positive symptoms and radiological findings but remain testing negative for SARS-CoV-2 RNA common respiratory (throat swab and sputum) specimens; they have features more similar to confirmed COVID-19 cases than non-COVID-19 pneumonia cases and may transmit SARS-CoV-2 to others. Such cases might need to be managed in the same way as confirmed COVID-19 cases, and add to the complexity and difficulty of COVID-19 control. Radiological examination might need to be further conducted for symptomatic SARS-CoV-2 RNA-negative contacts of COVID-19 cases. Our hypothesis-generating study highlights the importance of clinical diagnosis in this pandemic era, and suggests that SARS-CoV-2 RNA testing by rRT-PCR assays of common respiratory (throat swab and sputum) specimens, the widely accepted “golden standard” for diagnosing COVID-19, might be sometimes insufficient, and that further studies with some further procedures (e.g., testing via bronchoalveolar lavage or specific antibodies) would be warranted for Group 2-like patients, namely, the SARS-CoV-2 RNA-negative (tested using common respiratory specimens), radiographically positive, symptomatic contacts of COVID-19 cases, to further reveal their nature.

## Data Availability Statement

The datasets presented in this article are not readily available because access to the data needs to be requested with written proposals and protocols and to be approved by the corresponding authors and participating institutions. Requests to access the datasets should be directed to Aman Xu, amanxu@163.com.

## Ethics Statement

The studies involving human participants were reviewed and approved by the Ethics Committee of the Feidong People's Hospital, the eastern branch of First Affiliated Hospital of Anhui Medical University. The patients/participants provided their written informed consent to participate in this study. Written informed consent was obtained from the individual(s), and minor(s)' legal guardian/next of kin, for the publication of any potentially identifiable images or data included in this article.

## Author Contributions

LH, XZ, CZ, and AX had the idea for the study and full access to all data in the study and take responsibility for the integrity of the data and the accuracy of the data analysis. LH is an epidemiologist and physician scientist with clinical epidemiology as the major subject and public health and statistics as subjects during his PhD. XZ is an infectious disease specialist with rich work experience in isolation ward. LZ and JX have rich experience in clinical communication and data collection. YX is a professor of laboratory medicine. CZ is a respiratory medicine and critical care medicine specialist, and AX is a physician scientist with interest in clinical epidemiology and public health. LH, XZ, CZ, and AX played roles in the literature search, study conception and design, patient recruitment, clinical management, sample collection, data collection, analysis, interpretation, and writing of the report. LZ, JX, ZW, and YX played roles in the laboratory assays, data collection, analysis, interpretation, and confirmation and critical revision of the report. All authors contributed to the article and approved the submitted version.

## Conflict of Interest

The authors declare that the research was conducted in the absence of any commercial or financial relationships that could be construed as a potential conflict of interest.

## Publisher's Note

All claims expressed in this article are solely those of the authors and do not necessarily represent those of their affiliated organizations, or those of the publisher, the editors and the reviewers. Any product that may be evaluated in this article, or claim that may be made by its manufacturer, is not guaranteed or endorsed by the publisher.
